# Nutrition indicators as potential predictors of AIDS-defining illnesses among ARV-naïve HIV-positive adults in Kapiri Mposhi, Zambia 2008-2009

**DOI:** 10.1371/journal.pone.0219111

**Published:** 2019-07-02

**Authors:** Yi No Chen, Kristin M. Wall, Kadija Fofana, Carlos Navarro-Colorado

**Affiliations:** 1 Department of Epidemiology, Rollins School of Public Health, Emory University, Atlanta, Georgia, United States of America; 2 Division of Epidemiology and Biostatistics, School of Public Health, Georgia State University, Atlanta, Georgia, United States of America; 3 Division of Global Disease Protection, Center for Global Health, Centers for Disease Control and Prevention, Atlanta, Georgia, United States of America; Sefako Makgatho Health Sciences University, SOUTH AFRICA

## Abstract

Early changes in nutritional status may be predictive of subsequent HIV disease progression in people living with HIV (PLHIV). In addition to conventional anthropometric assessment using body mass index (BMI) and mid-upper arm circumferences (MUAC), measures of strength and fatigability may detect earlier changes in nutrition status which predict HIV disease progression. This study aims to examine the association between various nutritional metrics relevant in resource-scarce setting and HIV disease progression. The HIV disease progression outcome was defined as any occurrence of an incident AIDS-defining illnesses (ADI) among antiretroviral treatment (ART)-naïve PLHIV. From 2008–2009, HIV+ Zambian adult men and non-pregnant women were followed for 9 months at a Doctors without Borders (Medecins Sans Frontiers, MSF) HIV clinic in Kapiri Mposhi, Zambia. Since the study was conducted in the time period when former WHO recommendations on ART (i.e., ≤200 CD4 cell count as opposed to treating all individuals regardless of CD4 cell count or disease stage) were followed, caution should be applied when considering the implications from this study’s results to improve HIV case management under current clinical guidelines, or when comparing findings from this study with studies conducted in recent years. Bivariable and multivariable logistic regression was used to assess the associations between baseline nutritional measurements and the outcome of incident ADI. Self-reported loss of appetite study (AOR 1.90, 95% CI 1.04, 3.45, *P* = 0.036) and moderate wasting based on MUAC classification (AOR 2.40, 95% CI 1.13, 5.10, *P* = 0.022) were independently associated with increased odds of developing incident ADI within 9 months, while continuous increments (in psi) of median handgrip strength (AOR 0.74, 95%CI 0.60, 0.91, *P* = 0.004) was independently associated with decreased odds of incident ADI only among women. The association between low BMI and the short-term outcome of ADI was attenuated after controlling for these nutritional indicators. These findings warrant further research to validate the consistency of these observed associations among larger ART-naïve HIV-infected populations, as well as to develop nutritional assessment tools for identifying disease progression risk among ART-naïve PLHIV.

## Introduction

The relationship between malnutrition and HIV infection has been well documented in the existing literature [1, 2]. Because untreated HIV infection may impose a negative nutritional balance by increasing the static energy requirement and compromising nutrient intake and absorption, antiretroviral treatment (ART)-naïve PLHIV are an especially vulnerable group for acute malnutrition, which may leave patients with a greater risk of mortality or other serious health conditions (e.g., organ dysfunction) over a short period of time [3]. Although there has been tremendous progress in scaling up ART coverage worldwide in the last five years, a significant portion of PLHIV continues to remain ART naïve (40% in the WHO African region and 25% or about 275,000 PLHIV in Zambia as of 2017)[4, 5]. As food insecurity and subsequent malnutrition, prominent barriers to ART initiation and adherence [6], continue to plague many resource-scarce communities, understanding the prognostic relationship between nutritional indicators and short-term HIV disease progression among ART-naïve populations in those communities is critical.

Body Mass Index (BMI) and Mid-Upper Arm Circumference (MUAC) are often studied as indicators of malnutrition in adult patients, and have been associated previously with adverse clinical outcomes among PLHIV [7–15]. Several studies have shown a relationship between malnutrition related muscle fatigability and strength and adverse disease outcomes in HIV-free patients [16–19]. Furthermore, changes in muscular performance become observable before changes in weight and muscle mass when responding to both nutritional deprivation and nutritional repletion [20, 21]. This may suggest the potential utility of muscular strength and fatigability in predicting health outcomes among PLHIV who are at risk of HIV-associated malnutrition.

In light of the existing literature on the conventional nutritional predictors of poor HIV prognosis [7–14] and the growing evidence of other anthropometric measurements in describing the effect of malnutrition on health in various populations [16–19], the purpose of this 9-month prospective study was to assess the performance of a wide range of baseline nutritional indicators in predicting short-term HIV disease progression among ART-naïve adults in a severely resource-scarce setting. Since the study was conducted in 2008, the ART was recommended for PLHIV whose CD4 cell count drop below 200 cells/mm^3^ as opposed to all PLHIV regardless of CD4 and disease stage as per current WHO guidelines. Despite the potentially decreased relevance to current clinical management of HIV, we felt that the analysis may inform understanding of the prognostic capacities of common nutritional indicators among ARV-naïve PLHIV in certain resource-scarce areas where patients may not have immediate access to ART or patients delayed ART care. This may contribute to the development of field-friendly nutrition screening tools for the early identification of malnourished PLHIV at risk of hastened disease progression.

## Materials and methods

### Study location, participants, and design

Kapiri Mposhi is a primarily rural district in Central Province, Zambia. The town of Kapiri Mposhi lies on a major transportation route for Zambians and travellers from neighbouring countries. In 2006, the district had an estimated HIV prevalence of 17.4% [22].

From December 12, 2007 to January 30, 2009, HIV-positive adults aged ≥ 18 years who attended HIV clinics run by MSF-Operational Centre Barcelona and Athens (MSF-OCBA) in the town of Kapiri Mposhi, Zambia were enrolled in a 9-month prospective cohort study entitled “Early detection of nutritional status and prediction of survival in HIV patients: analysis of anthropometry and functional indicators” (IRB00001131). This study and the secondary analysis were approved by the Research Ethic Committee at University of Zambia and by the Office for Human Research Protections-registered Institutional Review Boards at Emory University, respectively.

Nutritional information was collected from enrolled patients using a questionnaire administered at study admission and at quarterly nutritional follow-up visits (3 and 6 month) (S1 Fig). MSF-OCBA HIV clinics used *Follow-Up of Clinical HIV Infection and AIDS* (FUCHIA *v*. *1*.*5*.*1*, Epicentre, Paris) software to collect and store demographic and clinical data from patients at their clinical visits. Patients from the clinics were actively referred to enroll in ART treatment programs if the 2006 World Health Organization (WHO) treatment criteria were met.

This analysis includes patients who were ART-naïve prior to and upon admission into the study who were strong enough to stand and be measured. Women who were pregnant and/or lactating were excluded, because they have meaningfully different metabolic characteristics that may require different nutritional assessment schemes than majority of our target adult population. The follow-up period was defined as the time between the study admission date and the date of ART initiation or the end of the 9-month study end date. Upon recruitment, patients who participated in the study would provide written consents. Since very few participants received any form of nutritional support at (n = 5) or after baseline (n = 6), the analysis aimed to target resource-scarce populations with no substantial external nutritional support programs in place.

### Outcome of interest

Incident ADI was the outcome of interest in this study. According to the WHO disease staging system for HIV infection and disease in adults and adolescents, the following clinical conditions are considered ADIs (i.e., severe HIV-associated symptoms, clinical stage 4 of HIV infection, and requiring ART initiation regardless of the patient’s immune status): *HIV wasting syndrome*, *Pneumocystis pneumonia*, *Recurrent severe bacterial pneumonia*, *Chronic herpes simplex infection (orolabial*, *genital or anorectal of more than one month’s duration or visceral at any site)*, *Oesophageal candidiasis (or candidiasis of trachea*, *bronchi or lungs)*, *Extrapulmonary and pulmonary tuberculosis (TB)*, *Kaposi’s sarcoma*, *Cytomegalovirus infection (retinitis or infection of other organs)*, *Central nervous system toxoplasmosis*, *HIV encephalopathy*, *Extrapulmonary cryptococcosis including meningitis*, *Disseminated non-tuberculous mycobacterial infection*, *Progressive multifocal leukoencephalopathy*, *Chronic cryptosporidiosis (with diarrhoea)*, *Chronic isosporiasis*, *Disseminated mycosis (coccidiomycosis or histoplasmosis)*, *Recurrent non-typhoidal Salmonella bacteraemia*, *Lymphoma (cerebral or B-cell non-Hodgkin) or other solid HIV-associated tumours*, *Invasive cervical carcinoma*, *Atypical disseminated leishmaniasis*, *Symptomatic HIV-associated nephropathy or symptomatic HIV-associated cardiomyopathy* [23]. Regardless of participants’ status for these symptoms prior to the study, those newly diagnosed with at least one of these conditions during the follow-up period were considered to have had the outcome of interest.

### Non-nutritional covariates

Baseline age, sex, CD4 cell count, diagnoses of non-severe HIV-associated symptoms, and diagnoses of ADIs were recorded at the time of entry into the study. Baseline age was trichotomized based on sex-specific tertile cut-points: 1) Male: age < 31 years/ Female: age years < 28 years; 2) Male: 37 years > age ≥ 31 years/ Females: 34 years > age ≥ 28 years; 3) Male: age ≥ 37 years/ Female: age ≥ 34 years. A dichotomous variable of baseline immunosuppression was made to indicate CD4 cell count lower than 200 cells/mm^3^.

Non-severe HIV-associated symptoms were coded as a composite binary variable describing a diagnosis of at least one of the following conditions: weight loss, minor mucocutaneous manifestations, herpes zoster, recurrent upper respiratory tract infections, bedridden during the last month, oral hairy leukoplakia, unexplained chronic diarrhea > 1 month, unexplained prolonged fever > 1 month, oral candidiasis, vulvovaginal candidiasis > 1 month [23]. The diagnosis of ADI at baseline was also a binary variable created to describe the diagnosis of at least one of the clinical stage 4 HIV-associated symptoms.

### Nutritional covariates

Loss of appetite and loss of weight were self-reported. Food security at the household level was assessed by asking whether participants experienced any of the following in the last three months prior to the visit: 1) being unable to eat the preferred types of food due to lack of resources; 2) having to eat a smaller or fewer meals than usual; 3) having no food stored in the participant’s household, and 4) sleeping on an empty stomach.

The techniques for performing MUAC, height, and weight measurements followed the standard Médecins Sans Frontières/Doctors Without Borders protocols. Briefly, MUAC was measured on the left arm, when the arm is relaxed at the side of the body, at the mid-point between the elbow and the shoulder. A non-stretchable measuring tape was placed around the arm and the measurement was read from the window of the tape without pinching the arm or leaving the tape loose. The MUAC was recorded to the nearest millimeter. Height was measured using an estadiometer, to the closest millimeter. Weight was measured using standing scales, to the closest 100 grams. Scales were calibrated daily using a known weight. BMI, MUAC, and height were measured by staff.

BMI was categorized into normal (BMI≥ 18.5), mild thinness (18.5 >BMI≥ 17.0), moderate thinness (17 >BMI≥ 16.0), and severe thinness (BMI < 16.0) using a grading system derived for adult chronic energy deficiency. Moderate and severe thinness were collapsed during regression analysis to obtain stable association estimates. MUAC was trichotomized according to sex-specific categories used in conditions of food scarcity: normal (Male: ≥ 230 mm; Female: ≥ 220 mm), moderate wasting (Male: 200–229 mm; Female: 190–219 mm), and severe wasting (Male: <200 mm; Female: <190 mm)[24]. Continuous BMI was excluded from the bivariable and multivariable modeling process, due to severely skewed distribution.

Muscular strength and fatigability were measured using two separate tests–the handgrip strength test and the “sphygmomanometer test”. The handgrip strength test required participants to squeeze a hand dynamometer 10 times consecutively with maximum force. Grip force (measured in psi) was recorded at each squeeze, and the following summary statistics were obtained from the ten handgrip force readings (in psi): median, slope, and percentage strength lost between the maximum reading and final reading. The two latter indicators were considered as measures of muscular fatigability and were calculated as follows:

$$Percent\;strength\;loss\;(\%)=\frac{\left(maximum\;reading-final\;reading\right)}{maxium\;reading}\times 100\%$$

$$Slope\;of\;measures=\frac{\left(final\;reading-maximum\;reading\right)}{\#trials\;between\;the\;maximum\;and\;final\;readings}$$

We devised the”sphygmomanometer test” to capture a composite measure of muscular strength and fatigability, by recording the cumulative pressure readings (mmHg) resulting from the repeated squeezes on the rubber bulb of mercury sphygmomanometer with maximum force for 60 seconds or until failure due to muscle fatigue. Each sphygmomanometer was emptied prior to conducting a new test. Cumulative pressure reading (mmHg) and the total number of hand squeezes obtained during the test were used to compute the average squeeze strength (mmHg/squeeze). A dichotomous variable was created to indicate if a participant failed at completing the 60-second “sphygmomanometer test”.

For all continuous measurements created from the two muscular strength and fatigability tests, skewness was obtained to assess distribution normality. Continuous measurements that had significantly skewed distribution (i.e., |skewness| > 0.50) were dichotomized using sex-specific median cut-point such that the characteristics could be modeled in logistic regression: percent strength loss (handgrip strength/fatigue test), slope of handgrip measures (handgrip strength/fatigue test), cumulative grip strength (“sphygmomanometer test”), and average grip strength (“sphygmomanometer test”).

### Data management and statistical analysis

Data cleaning and analyses were conducted using MS Excel, SAS v9.3 and SAS v9.4 (Cary, NC). The distributions of nutritional and non-nutritional characteristics were described by the outcome of incident ADI diagnosis using frequencies and percentages for categorical covariates or means and standard deviations for continuous covariates. Chi-square tests (for categorical variables), two-sample equal variance t-tests for normally distributed data (for continuous variables), and Mann-Whitney U for non-parametric data (for continuous variables) were used to compare the distribution of characteristics by the outcome incident ADI diagnosis. The characteristics that were originally continuous measurement but categorized based on cutoff percentile were treated only as continuous variables for descriptive analysis. The distributions of incident ADI by illness type and the sum of ADI incidence and related visits per person were described.

Logistic regression models were used to assess bivariate and multivariate associations between covariates and incident AIDS-defining illnesses. The nutritional covariates that had significant crude odds ratios (*P*<0.05) in the bivariate analyses were candidate exposure variables for inclusion in a multivariable logistic regression model, while all significant non-nutritional covariates were included as potential confounders. Some nutritional characteristics that were represented by both categorical and continuous variables, and if both were significant in the bivariable model, only the categorical variable would be included in a given multivariable model in order to maximize the number of covariates we can keep in the model.

To build the final multivariable logistic regression model, covariates that were candidates for the final multivariable logistic models were assessed for collinearity. When at least two covariates were associated with a Conditional Index greater than 30 and variance decomposition proportion greater than or equal to 0.5, one of the covariates was dropped. The decision about which covariate to drop was based on the statistical significance and clinical meaningfulness of the variable, as well as the overall fitness of the model. Dichotomous baseline immunosuppression was excluded from the final primary multivariable model, as its inclusion would have reduced the sample size (by 24%) and event count (by 29%) considerably. All exposure variables were also assessed for effect-modification by sex using likelihood ratio test. Adjusted odds ratio and 95% CI were obtained for each exposure in a final model. For the purpose of sensitivity analysis, two separate multivariable logistic models were run: 1) a multivariable model (adjusting for the same set of variables as in the final model) excluding participants who were diagnosed with ADI at baseline; 2) a multivariable model adjusting for the variables included in the final model, and baseline CD4, a variable that was excluded from the primary model due to missingness (24% of baseline CD4 results were missing in the analysis dataset). Due to insufficient sample size (n = 43), no additional multivariable model was performed for participants with baseline ADI.

## Results

### Baseline non-nutritional and nutritional covariates

The cohort was comprised of 238 men (42%) and 334 women (58%). During the nine-month follow up of the study, 28 men (12%) and 47 women (14%) were diagnosed with an incident ADI. Among the 75 participants who developed incident ADI, there were overall 79 ADI events (75 new illness and 4 recurrent illness). Throughout the study period, all cases had only 1 follow-up clinical visit where at least an incident ADI event was recorded. 71 cases had only 1 incident ADI event in that visit, while other 4 cases had 2 concurrent incident ADI events. On average, cases developed 1.05 incident ADI event over the 9-month follow-up. The two most prominent ADI in this cohort included pulmonary (58%) and extra-pulmonary (14%) TB, which together accounted for 72% of all incident ADI events.

Table 1 describes unstratified and stratified distribution of baseline characteristics by the outcome of interest. More than one-third of participants reported some degrees of food insecurity, ranging from inability to eat preferred good (42%) to sleeping with empty stomach (32%). About 13% and 7% of participants experienced moderate or severe thinness (i.e., BMI<17) and severe wasting according to MUAC at baseline, respectively. Participants experiencing an incident ADI during follow-up were more likely to have baseline immunosuppression (69% *v*. 45%, *P* = 0.002), self-report loss of appetite (72% *v*. 49%, *P*<0.001), have lower BMI (18.6 *v*. 20.0 kg/m^2^, *P*<0.001), and have lower MUAC (220.1 *v*. 243.8 mm, *P*<0.001) than those who did not experience an incident ADI. Additionally, those experiencing an incident ADI during follow-up were more likely to have BMI lower than 18.5 kg/m^2^ (49% *v*. 28%, *P*<0.001) and have MUAC lower than 230 mm for men or 220 mm for women (55% *v*. 26%, *P*<0.001). Compared to the sub-population that did not develop incident ADI, greater proportion of participants experienced moderate or severe thinness based on baseline BMI in those that developed incident ADI (26% *v*. 11%). Similarly, greater proportion of participants experienced severe wasting based on the baseline measurement of MUAC in the subpopulation that developed incident ADI, compared to those that did not (19% *v*. 5%).

**Table 1:** Baseline characteristics of HIV-positive adults in Kapiri Mposhi, Zambia, by the incidence of AIDS-defining illnesses (ADI), 2008–2009.

		*All (N = 572)*		*No incident ADI(N = 497)*		*Had incident ADI(N = 75)*		
		*n*	*%*	*n*	*%*	*n*	*%*	P^*^
**Non-nutritional covariates**								
Sex: Male		238	42%	210	42%	28	37%	0.420
Age**		32.9^†^	7.3^†^	33.1^†^	7.3^†^	31.9^†^	7.2^†^	0.186
Baseline CD4: *<200 cells/mm*^*3*^		209	48%	172	45%	37	69%	0.002
ADI at baseline: Yes		43	8%	37	7%	6	8%	0.865
Non-severe HIV-associated symptoms: Yes		225	39%	188	38%	37	49%	0.057
**Appetite and Weight Loss**								
Loss of Weight: Yes		455	80%	389	78%	66	88%	0.052
Loss of Appetite: Yes		299	52%	245	49%	54	72%	<0.001
**Household Food Security**								
Unable to eat preferred food		239	42%	211	43%	27	36%	0.318
Have smaller/fewer meals		239	42%	211	43%	26	35%	0.223
No food storage in household		186	33%	167	34%	19	26%	0.168
Sleeping empty stomach		184	32%	165	33%	19	26%	0.189
**Anthropometry**								
Continuous BMI(kg/m^2^)		19.8^‡^	3.6^‡^	20.0^‡^	3.7^‡^	18.6^‡^	3.1^‡^	<0.001
Categorical BMI								<0.001
*Normal*		398	70%	360	72%	38	50%	
*Mild thinness*		99	17%	81	16%	18	24%	
*Moderate thinness*		38	7%	27	5%	11	15%	
*Severe thinness*		37	6%	29	6%	8	11%	
Continuous MUAC (mm)		240.7^†^	33.3^†^	243.8^†^	32.5^†^	220.1^†^	31.3^†^	<0.001
Categorical MUAC								<0.001
*Normal*		398	70%	364	74%	34	45%	
*Moderate wasting*		131	23%	104	21%	27	36%	
*Severe wasting*		40	7%	26	5%	14	19%	
**Handgrip Strength Test**								
Median of measures (psi)		9.9^†^	3.0^†^	10.1^†^	3.0^†^	8.4^†^	3.0^†^	<0.001
Slope of measures**		-0.28^‡^	0.11^‡^	-0.25^‡^	0.11^‡^	-0.33^‡^	0.11^‡^	0.338
Percent strength loss (%)**		23.1^‡^	13.3^‡^	22.2^‡^	13.3^‡^	25.0^‡^	17.5^‡^	0.001
**Sphygmomanometer Test**								
Ability to complete the test		211	39%	178	38%	33	50%	0.057
Total number of grips		64.7^†^	16.9^†^	65.4^†^	16.9^†^	60.2^†^	16.2^†^	0.019
Average grip strength (mmHg) **		2.1^‡^	1.2^‡^	2.1^‡^	1.1^‡^	2.2^‡^	1.3^‡^	0.469
Cumulative handgrip strength (mmHg) **		150.0^‡^	30.0^‡^	150.0^‡^	30.0^‡^	140.0^‡^	50.0^‡^	<0.001

*for categorical variables, two-sided p-values from Chi-square tests; for continuous variables, two-sided p-values from t-test (if normally distributed) or Mann-Whitney U (if non-parametric)

**only the continuous variable of a baseline characteristic was shown in this descriptive statistics table, because their categorical counterparts were created based on the median or tertile values.

†mean and standard deviation were obtained for continuous variables with approximately normal distribution

‡ median and interquartile range were obtained for continuous variables with highly skewed distribution

Participants experiencing an incident ADI during follow-up had lower median (8.4 *v*. 10.1 psi, P<0.001) grip strength, had a higher percent handgrip strength loss (25.0% v. 22.2%, *P* = 0.001), produced smaller number of grips (60.2 v. 65.4 grips, *P* = 0.019), and had lower cumulative strength measures (140.0 *v*. 150.0 mmHg, *P*<0.001) on “sphygmomanometer test” kit.

### Bivariate and multivariate analysis

Significant bivariate associations were found between the diagnosis of incident ADI and ten covariates (Table 2). The following variables were included in the final multivariable logistic regression model: loss of appetite, categorical BMI, categorical MUAC, median of measures in handgrip strength test, categorical percent strength loss, total number of grip and categorical cumulative grip strength in the sphygmomanometer grip test. We observed negative bivariate trends between BMI and the odds of incident ADI (OR _mild_ 2.11, 95% CI 1.14, 3.88, *P* = 0.017; OR _moderate/severe_ 3.21, 95% CI 1.73, 5.97, *P*<0.001), and between MUAC and odds of incident ADI (OR _moderate_ 2.78, 95% CI 1.60, 4.82, *P*<0.001; OR _severe_ 5.77, 95% CI 2.75, 12.07, *P*<0.001).

**Table 2:** Unadjusted logistic model of incident AIDS-defining illnesses among HIV-positive adults in Kapiri Mposhi, Zambia, 2008–2009.

	*OR*	*95% CI*		*P*
**Non-nutritional covariates**				
Sex (n = 572, event = 75)				
*Female*	1.00	-	-	-
*Male*	0.81	0.49	1.34	0.421
Age (n = 572, event = 75)*				
*1st tertile*	1.00	-	-	-
*2nd tertile*	1.30	0.74	2.30	0.359
*3rd tertile*	0.72	0.38	1.38	0.326
Baseline CD4 cell count (n = 433, event = 54)				
*≥ 200 cells/mm3*	1.00	-	-	-
*< 200 cells/mm3*	2.62	1.43	4.82	0.002
Occurrence of AIDS-defining illnesses at baseline (n = 497, event = 54)				
*No*	1.00	-	-	-
*Yes*	1.08	0.44	2.66	0.865
Occurrence of non-severe HIV-associated symptoms (n = 572, event = 75)				
*No*	1.00	-	-	-
*Yes*	1.60	0.98	2.61	0.059
**Appetite and Weight Loss**				
Loss of Weight (n = 572, event = 75)				
*No*	1.00	-	-	-
*Yes*	2.04	0.98	4.22	0.056
Loss of Appetite (n = 572, event = 75)				
*No*	1.00	-	-	-
*Yes*	2.65	1.55	4.51	<0.001
**Household Food Security**				
Unable to eat preferred food (n = 569, event = 74)				
*No*	1.00	-	-	-
*Yes*	0.77	0.47	1.28	0.319
Have smaller/fewer meals (n = 569, event = 74)				
*No*	1.00	-	-	-
*Yes*	0.73	0.44	1.21	0.224
No food storage in household (n = 569, event = 74)				
*No*	1.00	-	-	-
*Yes*	0.68	0.39	1.18	0.170
Sleeping on empty stomach (n = 569, event = 74)				
*No*	1.00	-	-	-
*Yes*	0.69	0.40	1.20	0.191
**Anthropometry**				
Categorical BMI (n = 572, event = 75)				
*Normal*	1.00	-	-	-
*Mild thinness*	2.11	1.14	3.88	0.017
*Moderate/Severe thinness*	3.21	1.73	5.97	<0.001
Continuous MUAC (mm) (n = 569, event = 75)	0.98	0.97	0.99	<0.001
Categorical MUAC (n = 569, event = 75)				
*Normal*	1.00	-	-	-
*Moderate wasting*	2.78	1.60	4.82	<0.001
*Severe wasting*	5.77	2.75	12.07	<0.001
**Handgrip Strength/Fatigue Test**				
Median of measures (psi) (n = 551, event = 67)	0.81	0.74	0.89	<0.001
Categorical slope of measures (n = 551, event = 67)*†				
*1st 50th percentile*	1.00	-	-	-
*2nd 50th percentile*	1.46	0.83	2.56	0.190
Categorical percent strength loss (n = 551, event = 67)*†				
*1st 50th percentile*	1.00	-	-	-
*2nd 50th percentile*	2.37	1.39	4.05	0.002
**Sphygmomanometer Test**				
Ability to complete the test (n = 537, event = 67)				
No	1.00	-	-	-
*Yes*	1.65	0.98	2.76	0.059
Total number of grips (n = 548, event = 67)	0.98	0.97	1.00	0.020
Categorical average grip strength (n = 547, event = 66)*†				
*1st 50th percentile*	1.00	-	-	-
*2nd 50th percentile*	1.08	0.64	1.80	0.781
Categorical cumulative handgrip strength (n = 547, event = 66)*†				
*1st 50th percentile*	1.00	-	-	-
*2nd 50th percentile*	1.79	1.07	3.00	0.027

*categorical cutoff was made according to sex-specific median values

† treated as categorical variable in the logistic regression modeled due to the severely skewed distribution of the continuous measurement

The absence of meaningful collinearity among covariates in the multivariable logistic model was confirmed (Conditional Index = 29.5). The model suggested three independent predictors of incident ADI among HIV-positive Zambian adults (Table 3). Controlling for all other covariates, the odds of incident ADI among participants that self-reported loss of appetite at baseline was 1.90 times greater than those who did not (AOR 1.90, 95% CI 1.04, 3.45, *P* = 0.036); the odds of incident ADI among participants who experienced moderate wasting based on baseline MUAC measurements was 2.40 times (AOR _moderate_ 2.40, 95% CI 1.13, 5.10, *P* = 0.022) greater than those classified as normal. In addition, the association between the median of handgrip strength measures and the incident ADI was different by sex (likelihood ratio test for the interaction term: *P* = 0.009): among female participants, each one psi decreased in median handgrip strength on average was significantly associated with increasing the odds of developing incident ADI by 1.35 times (AOR 0.74, 95%CI 0.60, 0.91, *P* = 0.004), controlling for all other covariates. However, the median value of handgrip strength measures was not independently associated with the outcome of incident ADI among male participants (AOR 1.03, 95% CI 0.88, 1.22, *P* = 0.687).

**Table 3:** Multivariable logistic model of incident AIDS-defining illnesses among HIV-positive adults in Kapiri Mposhi, Zambia, 2008–2009 (N = 545, Event = 66).

	AOR*	*95% CI*		*P*
Loss of Appetite				
*No*	1.00	-	-	-
*Yes*	1.90	1.04	3.45	0.036
Categorical BMI				
*Normal*	1.00	-	-	-
*Mild thinness*	1.17	0.54	2.54	0.686
*Moderate/Severe thinness*	0.71	0.23	2.17	0.559
Categorical MUAC				
*Normal*	1.00	-	-	-
*Moderate wasting*	2.40	1.13	5.1	0.022
*Severe wasting*	3.79	0.92	15.6	0.065
**Handgrip Strength/Fatigue Test**				
Median of measures (psi)				
*Female*†	0.74	0.6	0.91	0.004
*Male*†	1.03	0.88	1.22	0.687
Categorical percent strength loss				
*1st 50th percentile*	1.00	-	-	-
*2nd 50th percentile*	1.32	0.71	2.45	0.384
**Sphygmomanometer Test**				
Total number of grips	0.99	0.97	1.01	0.311
Categorical cumulative handgrip strength				
*1st 50th percentile*	1.00	-	-	-
*2nd 50th percentile*	0.89	0.44	1.78	0.724

* adjusted for other covariates included in the multivariable model

† sex-specific effect presented due to significant effect modification by sex (i.e., interaction term with likelihood ratio test p-value<0.05)

### Sensitivity Analysis

After excluding the participants with baseline ADI, the associations between two of the predictors from the final multivariable model and incident ADI became mildly attenuated: loss of appetite (AOR 1.71, 95% CI 0.93, 3.17, *P* = 0.085) and MUAC (AOR_moderate_ 2.08, 95% CI 0.95, 4.55, P = 0.069; AOR_severe_ 3.47, 95% CI 0.72, 16.88, *P* = 0.1226). On the other hands, the association between median handgrip strength and the study outcome remained relative the same in the effect size and continued to be statistically significant among women (AOR 0.73, 95%CI 0.60, 0.89, P = 0.003).

After adjusting for baseline CD4, the associations between all three former predictors and short-term ADI became mildly attenuated in effect size: median handgrip strength among women (AOR = 0.77, 95% CI 0.61, 0.95, *P* = 0.020), loss of appetite (AOR = 1.73, 95% CI 0.86, 3.49, *P* = 0.124), and MUAC (AOR_moderate_ 2.17, 95% CI 0.89, 5.27, P = 0.087; AOR_severe_ 2.59, 95% CI 0.49, 13.59, P = 0.262). To ensure the absence of multicollinearity in the CD4-adjusted model, we excluded cumulative handgrip strength (as per the “sphygmomanometer test”) from the controlling covariates.

## Discussion

Our analysis sought to understand if common nutritional indicators measured in a resource-scarce clinical setting had meaningful prognostic value for HIV disease progression among ART-naïve PLHIV. This may inform and potentially improve case management of HIV patients in resource-poor or emergency contexts, where co-morbidity with malnutrition may be prevalent. Our primary multivariable model suggested the following three baseline nutritional indicators were independently associated with the short-term elevated odds of incident ADI: self-reported loss of appetite and low MUAC measurements among PLHIV of both sexes and median handgrip strength measures among women. The findings from sensitive analysis were overall confirmatory in the estimated effect sizes, however, with less statistical power to validate the true association between the three nutritional indicators and the study outcome. Since about ¾ of the incident ADI events were related to TB, our study may be more relevant to describing relationship between malnutrition and TB-related ADI. The TB-heavy distribution found in this study is consistent with the knowledge that TB represented more than half of the AIDS-defining conditions in many developing countries, as well as the epidemiologic context of high HIV/TB comorbidity in Sub-Saharan Africa [25, 26].

The observed association with self-reported appetite loss in this study (AOR 1.90, 95% CI 1.04, 3.45, *P* = 0.036) is consistent with the existing body of evidence in which reduced appetite has been linked with poor nutrition and worsened HIV disease progression and treatment outcomes [27–29]. Such an association independent of other well-recognized nutritional prognostic markers (e.g., BMI, MUAC) could imply the potential utility of appetite loss assessment in screening ART-naïve PLHIV at risk of hastened disease progression in areas where access to adequate clinical and nutritional assessment is limited. On the other hand, the researchers from the Nutritional Support for Africans Starting Antiretroviral Therapy (NUSTART) trial [30] only observed crude association between mortality and baseline appetite among Tanzanian and Zambian PLHIV during the follow-up period between ART referral and early treatment phase [31]. This could potentially imply that recent appetite loss, which reflects change in appetite to some degree, may be better predictor of acute disease progression than a one-time measurement of appetite. Nevertheless, caution should be applied when interpreting the observed association between appetite loss and incident ADI, considering the TB-heavy distribution in our study population. It is possible that the association could be confounded by the already-existing yet undiagnosed TB disease, which commonly induces loss of appetite [32]. NUSTART and another randomized control trial also suggest a sizable burden of undiagnosed TB among malnourished Zambian PLHIV [31, 33]. From this perspective, increasing TB interventions (e.g., diagnosis and treatment) could potentially benefit ARV-naïve PLHIV who experienced malnutrition in rural Zambia.

Although various measures of weight loss and food insecurity have been shown to predict worsened HIV disease progression in some studies [34–36], we did not observe crude nor adjusted associations between short-term incident ADI and these self-reported variables in our study. It is possible that the lack of association with food insecurity could be related to the use of non-standard food security questionnaire. It could also imply the presence of recall or response biases [37]. For example, patients may over-report the events of food insecurity if they perceive food aids dependent on their responses [38]. Self-reported weight loss, on the other hands, relies on patients’ memory of their usual weights, which could be inaccurate for people who are not accustomed to tracking their own weight and/or do not have regular access to proper equipment. Self-reported loss of appetite is subjective and may also be subjected to recall bias [39]. However, the observed difference in the predictive associations among these three self-reported variables potentially suggest that self-reported loss of appetite may have been a more robust or objective measure than weight loss and food security.

A cross-sectional study done by Kelly P et al. in Zambia has suggested that low MUAC and BMI measurements were associated with the presence of opportunistic infection, a study outcome that included many clinical manifestation of ADI among ART-naïve PLHIV [9]. Our findings further supplement the evidence on the linkage between MUAC and clinical markers of HIV/AIDS among ART-naïve PLHIV by demonstrating that moderate wasting (based on baseline MUAC measurement) independently predicted short-term incident ADI after controlling for various nutritional metrics. In addition, the finding of a survival analysis study done among ART-naïve HIV patients in National Simão Mendes Hospital (NSMH) of Guinea-Bissau suggests that low MUAC (i.e., ≤ 250 mm) independently predicts mortality within 2.5 years among ART-naïve PLHIV, after adjusting for sex, HIV type, CD4 and BMI [40]. This could potentially imply that MUAC is not only an independent predictor for short term adverse clinical outcome, indicated by incident ADI within 9 months, but also for relatively long term and more severe disease outcome among ART-naïve PLHIV.

On the other hand, the observed association between BMI and the short-term ADI was significantly attenuated in the multivariable model. This is rather interesting, because MUAC, which has been shown strongly correlated with BMI among various populations [41, 42] including our study participants (chi-square test between categorical MUAC and BMI, *P*<0.001; correlation coefficient between continuous MUAC and BMI = 0.851, *P*<0.001), remains independently predictive of the short-term ADI. This could imply that MUAC is a better prognostic marker for hastened disease progression among ART-naïve PLHIV. This nuance between MUAC and BMI may be consistent with the findings of a nutritional supplementation trial among 1561 emergency care patients, in which BMI failed to predict mortality adjusting for MUAC and weight loss while MUAC remained predictive [43]. Further studies with larger sample size may still be required to validate the attenuation of the association between BMI and hastened disease progression after controlling for MUAC, as past studies have shown BMI to be independently predict worsened HIV disease progression over longer follow-up period [8, 44, 45]. Nevertheless, the relatively easy measurement of MUAC in resource-scarce clinical setting and for physically disable patients renders it great potential for field use as possible prognosis tool [46, 47].

In addition, our multivariable model shows a statistically marginal association between severe wasting and incident ADI. The greater adjusted OR could potentially imply that the association with short-term incident ADI may be even stronger for those with extreme degree of wasting as indicated by MUAC. However, given the limited sample size of participants with severely low MUAC, our study may not statistically validate such an implication; further research with ample samples on extremely low MUAC population may be required.

The predictive association observed among female PLHIV in this study is consistent with the growing body of epidemiologic narratives which demonstrates handgrip strength, a common index for functional status, as a prognostic marker for short-term and long-term adverse health outcomes (e.g., hospitalization, premature death, physical frailty) in various populations [20]. A nutritional supplementation trial in Tanzania and Zambia similarly demonstrates that low handgrip strength is associated with elevated risk of mortality among ART-naïve PLHIV, independent of BMI [31]. The observed difference in the association between median handgrip strength and incident ADI by sex may imply possible effect measure modification by sex. However, the validity of such implication remains questionable due to lack physiological basis in the current literature. Limited studies that assess handgrip strength as a predictor of adverse health outcomes report on effect measure modification by sex. A study of 35–74 year-old Adult Health Study (AHS) cohort members in Japan shows significant difference in the association between low handgrip strength and mortality by sex—low handgrip strength (compared to the sex- and age-specific third quintile) was associated with higher risk of mortality in women aged between 35 and 54, but not in men [48]. However, the same study also reveals that the association between 5kg-increment of grip (which is similar to the continuous handgrip strength measurement used in this study) and all-cause mortality does not differ significantly by sex. It is possible that the observed difference in the effect measure by sex in this study may be due to the differential manifestations of malnutrition between men and women.

A primary limitation in this study is that we did not control for baseline immunosuppression (i.e., CD4 count < 200 cells/mm^3^), a common predictor for HIV advanced disease progression [46], in our primary analysis due to significantly reduced sample size and event count. In the sensitivity analysis where we adjusted for baseline CD4, the associations of incident ADI with MUAC and with loss of appetite no longer reached statistical significance. This was likely in part due to loss of study power when controlling for baseline CD4. Even if the associations between incident ADI and the three nutritional predictors in the final model were truly confounded by baseline CD4, the results of this study may still be of great relevance for informing the clinical practices of identifying HIV patients at risk of hastened disease progression in settings where clinicians may not have sufficient laboratory resources to measure CD4 cell counts. In addition, we did not routinely collect data on participants’ socioeconomic status, previous events of ADI prior to the study period, or time of HIV infection. The inability to control for such characteristics may allow for residual confounding. The small sample size of the participants with baseline ADI did also not allow us to conduct a complete stratified assessment by baseline ADI. This study was conducted when WHO guidelines recommended PLHIV whose CD4 cell counts drop below 200 cells/mm^3^ initiate ARVs. With the recent ARV guideline of “Test and Treat” issued by WHO since 2015 [49, 50], we may expect a substantial decrease in the number of tested PLHIV who remain ARV-naïve in rural sub-Saharan Africa. This trend may affect the clinical applicability of our study findings in non-resource scarce regions. Lastly, weight loss, appetite loss, and household food security were self-reported and therefore subject to information bias.

The programmatic implications of this analysis may include providing nutritional supplements to HIV-infected populations at risk of malnutrition, which may impact both weight gain and treatment adherence [51]. A similar suggestion has also been made in one of the NUSTART trial studies, where vitamin and mineral supplementation was shown to improve CD4 count among PLHIV starting ART [49]. Other implications may include conducting further research to develop cost-effective HIV prognostic tools using a set of nutritional measurements. With the 2015 “Test and Treat” ARV initiation recommendation, the size of the eligible populations is expected to significantly increase worldwide [49]. This increased demand in ARV could aggravate the issue of sporadic drug shortage. In Zambia, about 24% of the PLHIV reported to have experienced ARV supply shortage from time to time according to a cross-sectional questionnaire conducted in Southern province in 2017 [52]. Moreover, about 39% of HIV-infected Zambians still were not retained in ART care 12 months after initiating the universal testing and treatment intervention based on the 2015 WHO guideline [53]. As a result, ensuring uninterrupted supply of ARV becomes a primary programmatic focus particularly in resource-scarce regions where sporadic drug supply and malnutrition may be more prevalent. Developing such a prognostic tool for acute disease progression could potentially encourage efficient allocation of medical resources and attention in those regions.

### Conclusion

The results of our analysis contribute to the growing body of knowledge that emphasizes the clinical importance of nutritional maintenance among PLHIV living in resource-scarce setting. Based on our findings, loss of handgrip strength for women, self-reported loss of appetite, and low MUAC independently predicted short-term incident ADI in ART-naïve adults in rural Zambia. Given the minimal requirement for measurement equipment, screening for low MUAC and self-reported loss of appetite may have practical values for potential field application in resource-poor clinics. One limitation of this study was that the power to statistically validate the association between the three nutritional indicators and incident ADI decreased after adjusting for baseline CD4 or excluding participants with baseline ADI. As a result, further research should be conducted on these nutritional indicators with larger sample size to validate their prognostic relationship with disease progression, as well as examine the presence of effect measure modification by sex.

## Supporting information

S1 Fig Original questionnaire used by MSF to assess nutritional status among HIV Zambians in Kapiri Mposhi, 2008–2009. (PDF)
