# Supplementary material for: Nutrition indicators as potential predictors of AIDS-defining illnesses among ARV-naïve HIV-positive adults in Kapiri Mposhi, Zambia 2008-2009
Source: PLoS One. 2019 Jul 2;14(7):e0219111. doi: 10.1371/journal.pone.0219111 (PMC6605674; doi:10.1371/journal.pone.0219111)
Supplement: S1 Fig — (PDF) [file pone.0219111.s001.pdf]

S1 Fig. Original questionnaire used by MSF to assess nutritional status among HIV Zambians in Kapiri Mposhi, 2008 – 2009

MSF-Spain Kapiri study. Nutritional status and HIV.

Nurse: ___________________ **Patient's FUCHIA number:** [ ]

Visit (circle): admission / start ARV / month 3 / month 6 / in hospital **Date:** _____/_____/_____

1. Anthropometry:

Age (years): ___________ Weight (kg): ________________ Height (cm): ____________________

Sex (M/F): ____________ MUAC (mm): _______________ Triceps skin-fold (mm): ___________

2. Appetite and Weight loss:

Has the patient presented recently any **loss of appetite** ? Y / N **When ?** __________________________

What is the **usual weight** of the patient ? _________ OR not known

Has the patient **lost weight** ? Y / N **Since when ?** ________________

3. Hand-grip strength (psi):

1	2	3	4	5	6	7	8	9	10

4. Activities of Daily Living:

Mark which of the following the patient IS NOT able to do WITHOUT HELP ?

**1**	**work in the fields or other heavy works**	**4**	**maintain continence and use a toilet**
**2**	**do his/her usual work**	**4**	**transfer from bed to chair**
**3**	**do basic work at home**	**5**	**wash and groom**
**3**	**go shopping or to market**	**5**	**walk**
**4**	**feed him/herself**	**5**	**stand**

ALL OK: _______

**During the last month, how many days you were not able to work because of being sick or too tired ?**	
**During the last month, how many days did you stay in bed sick ?**	

5. Baseline food security information: Mark with a cross in the relevant square (0=NO, 1=At least once)

In the past FOUR WEEKS, did/were you or any other HOUSEHOLD member...	0	1

1.... not able to eat the kinds of food you preferred (due to lack of resources) ?		
2.... have to eat a smaller or fewer meals than usual (because there was not enough food) ?		
3.... was there ever no food to eat in your household (due to lack of resources) ?		
4.... go to sleep with an empty stomach (because there was not enough food) ?		

Are you currently receiving any nutrition support (circle): MSF / DAPP / WFP / Hospital / Other:________

**6. Sphingomanometer test: Time (seconds): ________ Strokes: _________ Top reached (mmHg): ___________**

7. Offer the patient 1 tablet of BP100. How much can the patient eat ? __________________________ %
